# Doppler ultrasonography and exercise testing in diagnosing a popliteal artery adventitial cyst

**DOI:** 10.1186/1476-7120-7-23

**Published:** 2009-05-27

**Authors:** Maurizio Taurino, Luigi Rizzo, Nazzareno Stella, Massimo Mastroddi, Fabio Conteduca, Claudia Maggiore, Vittorio Faraglia

**Affiliations:** 1 Department of Vascular Surgery, University of Rome La Sapienza-, II Medical School, S. Andrea Hospital, Roma, Italy; 2 Kirk Kilgoura Sport Injuries Center, University of Rome a La Sapienza-, II Medical School, S. Andrea Hospital, Roma, Italy

## Abstract

We describe popliteal arterial adventitial cystic disease which causes intermittent claudication in a young athletic man, with atypical manifestation, without loss of foot pulses on knee flexion nor murmur in the popliteal fossa. The findings obtained from Magnetic Resonance Imaging were non-diagnostic. The diagnosis resulted from Echo-Doppler ultrasonography along with peak exercise testing. Ultrasonography also provided useful physiopathological informations suggesting that a popliteal artery adventitial cyst can become symptomatic if muscle exertion increases fluid pressure within the cyst, enough to cause hemodynamically significant endoluminal stenosis. Rapid diagnosis is essential to prevent progressive claudication threatening limb viability. To guarantee this professional sportsman a reliable and durable outcome, instead of less aggressive management, we resected the involved arterial segment and interposed an autologous saphenous-vein graft.

## Background

Popliteal arterial adventitial cystic disease is a rare disorder causing intermittent claudication that is well described in young adults, and was first reported by Atkins and Key [1] in a 40-year-old man. Most patients are males in their 40s or 50s who present with intermittent claudication of sudden onset and rapid progression.

In patients with advanced disease, the diagnosis generally rests on evidence of segmental popliteal artery stenosis or occlusion without other popliteal artery disease. Traditional angiography or more recently gadolinium-enhanced magnetic resonance imaging (MRI) angiographic scans disclose an eccentric popliteal artery narrowing known as the "scimitar" sign. In some cases, stenosis is visible only on images obtained during knee hyperflexion.

Although the precise etiology remains unknown, now the most accepted mechanism involves cystic degeneration causing vessel stenosis or occlusion without superimposed thrombosis. Increased pressure from the liquid within the cyst is thought to cause popliteal stenosis through an "intermittent" mechanism induced by physical exercise.

In the 34-year-old man we report here, since MRI angiography failed to visualize the cyst clearly, the definitive diagnosis of popliteal arterial adventitial cystic disease came from the ECHO-Doppler ultrasound examination.

## Case report

We describe here the case of a 34-year-old man, a professional football player in excellent physical conditions, with no clinical history of importance except traumatic rupture of the anterior cruciate ligament, the right in 1985 and the left in 1995, treated with reconstruction using the central third of the patellar tendon. The patient presented with the abrupt onset of intermittent claudication in the right calf after walking on the flat for 300–400 meters.

An Echo-Doppler ultrasound examination disclosed a suspected "small aneurysm" or "popliteal adventitial cyst" but no popliteal artery disease was seen on MRI angiography even on images obtained during forced knee flexion.

**Figure 1:**
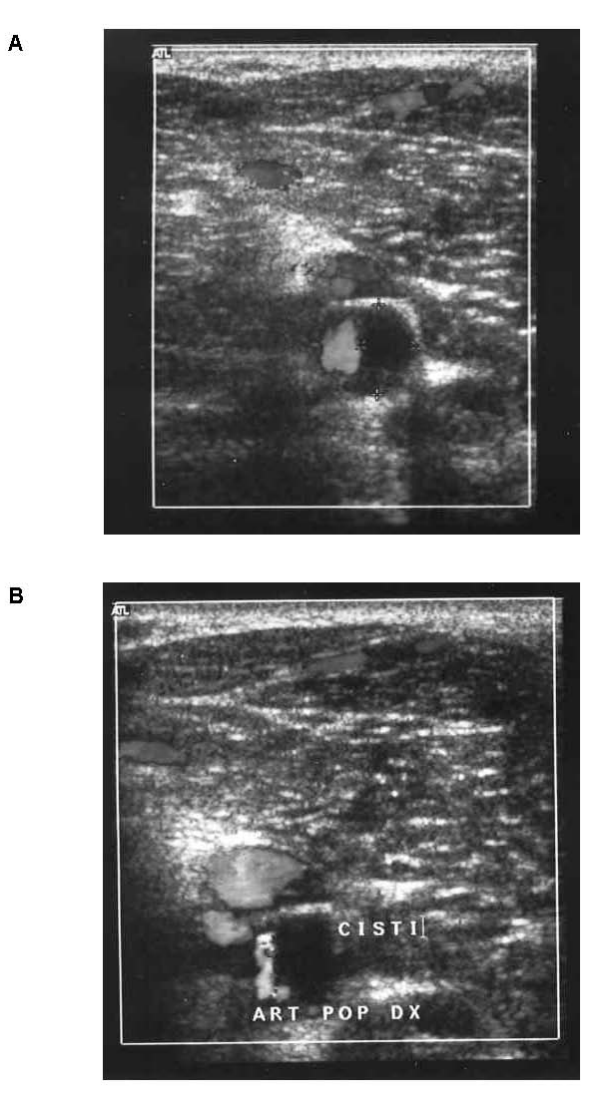
**Echocolor Doppler imaging showing narrowing of the popliteal artery lumen A) before exercise and B) after exercise**.

On the patient's admission to our vascular service, an Echo-Doppler ultrasound examination, obtained during hyperflexion, disclosed no peripheral perfusion deficit. On treadmill exercise testing, after 500 mt. the incline was increased from 10% to 15% and the speed from 6 km/h to 8.6 km/h. About 19 minutes later (about 1900 mt.) the patient began to experience pain in the right foot and, after exercising for a further 250 mt. with the incline at 12% and the speed at 7 km/h, he complained of cramp-like pain in the right calf. At peak force, and during cramp, the Echo-Doppler ultrasonogram showed an accentuated stenosis that involved the lumen of the right popliteal artery and corresponded to an increase in cystic volume. These changes were not discernible during baseline testing. (Fig. 1)

**Figure 2:**
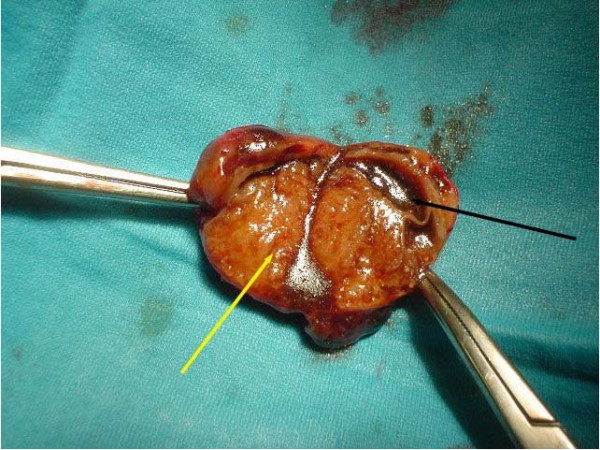
**Specimen of the adventitial cyst**. Please note the mucoid degeneration (yellow arrow) and the residual lumen of the artery (black arrow).

At surgery, the popliteal artery segment containing the cyst was resected (Fig. 2) and a reversed autologous great saphenous vein graft was interposed, through a posterior popliteal approach with a "bayonet" skin incision.

The histologic examination showed within the arterial adventitia an inflammatory mononuclear lymphocyte infiltrate, and a slightly thickened media made up of smooth-muscle cells with pyknotic nuclei and vacuolized cytoplasm, in a setting of myxoid degeneration that profusely involved the subintimal layer.

The patient had an uneventful postoperative course and, at a 15 months follow-up, he was asymptomatic.

## Discussion

A distinctive feature in this report is that our patient's popliteal artery adventitial cyst lacked both the currently accepted pathognomonic diagnostic signs: the loss of foot pulses with knee flexion (Ishizawa sign) [2] and the presence of a popliteal fossa murmur reported by Eastcott [3]. The most likely explanation is that because the cyst occupied only a small posteromedial segment of the popliteal artery it caused no endoluminal compression.

Particularly remarkable features in this report are the Echo-Doppler ultrasonographic findings clearly documenting the pathophysiological mechanism through which the popliteal artery adventitial cyst became symptomatic. Although previous investigators [4-8] described both the reduced distal pressure at the ankle, as measured on Doppler ultrasonography, and the angiographic features of popliteal stenosis after a symptom-provoking exercise test, they left the possible underlying mechanism unexplained. Our case report now suggests that an adventitial cyst of the popliteal artery can become symptomatic during muscle exertion when pressure within the fluid-filled cyst increases sufficiently to provoke hemodynamically significant endoluminal stenosis.

In cases like this reported here, ultrasounds examination can be more accurate than imaging techniques such as CT scan or MRI; Moreover ultrasounds are less expensive, widely available and free of ionsiong radiations.

This possible mechanism is supported from observations, unlike the case we report, describing cysts communicating with the synovial structures in the knee. Numerous reports describing treated Baker's cysts, that ultimately recurred, underline the need for meticulous surgical dissection of the cyst away from the adjacent joint structures in these cases and, also, in similar cases of cysts developing contiguously with the hip joint [9]. Arguing in favour of this mechanism of recurrence are the histopathological features of the lesion, postulating that a communication allows mucus-secreting cells to enter the adventitia. Notwithstanding the absence of visible communications with the adjacent joint structures, in the case we describe here, the histopathologic examination confirmed the presence of myxoid secreting cells.

Our decision to manage our patient's cyst by resecting the involved arterial segment and interposing an autologous saphenous vein graft, rather than undertaking CT- or ultrasound-guided drainage or surgical evacuation, depended primarily on the need to guarantee a young adult with a professional sports career a reliable and durable outcome.

## Conclusion

In spite of their rare occurrence, popliteal artery adventitial cysts are a disabling disease especially because they typically affect young otherwise healthy athletic adults. The diagnosis is usually reached by MRI angiography at rest. Even though arterial stenosis can become symptomatic and therefore visible on ultrasonography only after maximal prolonged exercise testing, ultrasonography is diagnostically far more reliable than more traditional imaging modalities. Popliteal arterial adventitial cystic disease should be managed surgically without delay, before the artery occludes, and radical surgery is essential to avoid the known risks of recurrence.

## Competing interests

The authors declare that they have no competing interests.

## Authors' contributions

MT and NS permormed the surgery and participated in the design of the study, LR and MM conceived the study and wrote the manuscript, FC gave orthopaedic contribution to the operation, CM performed the ultrasound test with VF.

## Consent

Written informed consent was obtained from the patient for publication of this case report and any accompanying images. A copy of the written consent is available for review by the Editor-in-Chief of this journal
